# Expectations of Reciprocal Generosity Are Specific to Equal Relationships

**DOI:** 10.1162/OPMI.a.357

**Published:** 2026-06-02

**Authors:** Alicia M. Chen, Rebecca Saxe

**Affiliations:** Department of Brain and Cognitive Sciences, Massachusetts Institute of Technology, Cambridge, MA, USA

**Keywords:** social cognition, generosity, reciprocity, social relationships

## Abstract

When someone acts generously, what expectations does this create? Classic theories and experiments emphasize reciprocity: the idea that generosity will be returned by the recipient. Yet daily-life and ethnographic observations often show a different pattern: generosity sets a precedent, leading people to expect the same person to be generous again. Across six online behavioral experiments (total *N* = 599 U.S. adults) using third-party vignette judgments and first-person incentivized economic games, we test when generosity creates expectations of reciprocation versus a precedent. We found that people expected reciprocity only in equal or symmetric relationships. Otherwise, they expected generosity to continue from the same actor. These expectations generalized across roles, contexts, and cost structures. Taken together, the results suggest that classic evidence on reciprocity and turn-taking may capture expectations among strangers or equals, but not the wider set of relationships that structure much of social life.

## INTRODUCTION

Two colleagues, Igor and Marie, go out for coffee, and Igor picks up the check. Who will pay the next time they get coffee? For many theorists in social and moral psychology (Cialdini, 2009; Earp et al., 2021; Gouldner, 1960; Le Pargneux & Cushman, 2025; Molm et al., 2007; Thibaut & Kelley, 1959), the answer is obvious: Marie. Acts of generosity are assumed to generate strong norms and expectations of reciprocity. When one person acts generously, the other is expected to return the favor. Such expectations can strongly influence future behavior, even outweighing personal preferences and self-interest (Bicchieri, 2005; Cialdini & Goldstein, 2004; Prentice & Paluck, 2020).

Here, we suggest that this classic assumption may not generalize to the broader landscape of human social relationships. Drawing on relational theories from anthropology and social psychology (Clark et al., 2017; Fiske, 1992; Graeber, 2011; Yan, 1996), we argue that direct reciprocity only governs a subset of social interactions: those between equals or strangers. Otherwise, generosity creates a precedent, rather than a debt that needs to be repaid. In other words, if Igor is Marie’s boss, his paying once may intuitively set the expectation that he will pay the next time as well. The present research examines when and why people might have these contrasting expectations, by applying a cognitive science approach to causally manipulate and measure how social-relational structure shapes the expectations created by a single generous act.

Decades of economic and psychological theories assume that a generous act typically elicits expectations of direct reciprocation. People who act generously anticipate repayment, and recipients often feel obligated to reciprocate (Blau, 1964; Cialdini, 2009; Flynn & Yu, 2021; Gouldner, 1960; Kardas et al., 2018; Trivers, 1971). Reciprocation alleviates indebtedness (Cialdini, 2009), fosters trust and positive affect (Molm et al., 2007), and maintains cooperation in repeated interactions (Axelrod & Hamilton, 1981; Fehr & Fischbacher, 2003; Rand & Nowak, 2013). In experimental incentivized games, partners often alternate giving and receiving roles (McKelvey & Palfrey, 2001; Prisbrey, 1993), a pattern observed even in children (Grueneisen & Tomasello, 2017; Melis et al., 2016; Olson & Spelke, 2008) and preverbal infants (Tatone & Csibra, 2020).

Yet anthropological and sociological accounts suggest that acts of generosity create expectations of reciprocation only in a narrow subset of social contexts—specifically, among social equals. For example, Graeber (2011) argues that reciprocal exchange implies the potential and desire for formal equality between partners, creating and sustaining relationships between people of equal rank in society. As a result, directly repaying generosity may be socially *inappropriate* when it could disrupt the norms that maintain a social hierarchy (Parry, 1986; Yan, 1996). Hierarchical relationships, Graeber speculates, instead function based on a logic of precedent: when someone acts generously, it creates the expectation that the same person will act generously again. For instance, ethnographic work from rural China shows that villagers routinely offer gifts to superiors with no expectation of return, to preserve the relationship and fulfill socially defined roles (Yan, 1996). In other words, in relationships that are already unequal, repeated acts of generosity may sustain the relationship precisely by *not* generating expectations of alternation or reciprocation.

More generally, anthropologists and sociologists conceptualize generous acts, like other social interactions, as a means to create and sustain different kinds of social relationships (Carrier, 1991; Fiske, 1992; Graeber, 2011; Mauss, 1925; Rai & Fiske, 2011; Sahlins, 2013; Schwartz, 1967; Weiner, 1992). Indeed, relationship structure shapes expectations about prosociality from early in development: infants and children typically expect reciprocation (Tatone & Csibra, 2020, 2024) but also coordinate along dominance asymmetries (Grueneisen & Tomasello, 2017), and both adults and children associate hierarchy with distinct patterns of responsibility and prosociality (Gopnik et al., 2025; Stavans & Diesendruck, 2021; Terrizzi et al., 2020). Yet the specific prediction that hierarchical relationships evoke expectations of *precedent*—articulated most explicitly by Graeber (2011), who supports it with observational evidence but which is really a speculation about the underlying human cognition—has not been experimentally tested.

Here we systematically tested this prediction, by examining the minimal conditions that influence the expectations created by a single generous act, for the next similar interaction between the same people. Specifically, when do people expect that the same individual will perform the generous role again (precedent), rather than expecting the recipient to play the generous role in return (reciprocity)? Consistent with most previous experimental research (McKelvey & Palfrey, 2001, Prisbrey, 1993), we recruited participants from a large-scale society with high market integration, the United States, where people have strong norms of balanced exchange (Henrich et al., 2005; Henrich, Ensminger, et al., 2010). By contrast to most previous experiments, we additionally provided minimal verbal descriptions contextualizing the interactions in hypothetical structured social relationships that were either equal or hierarchical. Across experiments, relationships were described as “symmetric” versus “asymmetric,” as one party having “equal,” “more” or “less power, status or influence” than the other, or using familiar concrete relationship labels like “cousins” or “friends,” versus “uncle-nephew” or “boss-employee.”

We hypothesized that expectations of direct reciprocity would be restricted to relationships with the potential or desire for equality—such as friends, neighbors, co-citizens, or anonymous strangers. In four experiments, we systematically measured third-party observers’ expectations for everyday interactions, and isolated the contribution of minimal relational information to these expectations. Then, in two experiments, we tested whether these findings generalize to participants’ own strategies in incentivized experimental games.

The verbal descriptions of relationships exerted strong and systematic effects on people’s expectations in all experiments. That is, even in large-scale societies where expectations of reciprocity and balanced exchange have been treated as a default, the expectations created by a single act of generosity differ substantially in structured social relationships.

### Implementation and Analysis Details

Experiments were implemented using the jsPsych library (de Leeuw et al., 2023), and analyses were conducted in R using the lme4, lmerTest, and emmeans packages (Bates et al., 2015; Kuznetsova et al., 2017; Lenth et al., 2018). For studies 3, 5, and 6, we used the DataPipe tool to automatically send the data to the Open Science Framework (de Leeuw, 2024).

For Studies 1–3 (linear mixed-effects models), we report Cohen’s *d* for pairwise contrasts, computed using the residual standard deviation from the model as the standardizer (Lenth et al., 2018). For Studies 4–6 (logistic mixed models), we report odds ratios with 95% Wald confidence intervals.

Where key theoretical conclusions are based on null findings, we supplement the frequentist tests with Bayes factors (BF_01_), computed using the BayesFactor package (Morey & Rouder, 2024) with the default JZS prior (Cauchy scale *r* = $\sqrt{2}$/2 for fixed effects; Rouder et al., 2012), to quantify the evidence in favor of the null hypothesis. BF_01_ expresses how many times more likely the data are under the null hypothesis than the alternative; values above 3 are conventionally interpreted as moderate evidence for the null, and values above 10 as strong evidence (Lee & Wagenmakers, 2014).

Sample sizes were determined in advance based on visual inspection of pilot data: once a sample size was chosen for Study 1, it was kept consistent for Studies 1–4, and similarly for Studies 5–6. Sensitivity analyses computing the minimum detectable effect sizes at 80% power for all studies are reported in the Supplementary Material; these use a paired-comparison framework as a conservative approximation to the full mixed-model structure.

## STUDY 1: BEING GENEROUS CREATES A PRECEDENT IN HIERARCHICAL RELATIONSHIPS

First, we tested participants’ third-party evaluations of scenarios describing characters playing the generous role in daily-life interactions (e.g., buying coffee during a work meeting, preparing a meal and cleaning after, deferring to the other’s preference; see Supplementary Material Table S2 for all the 18 scenarios), given information about a social relationship between the two characters. Here we use the term ‘generous’ to describe any social interaction between two people in which one person bears more cost, or receives less benefit, than the other. This definition includes interactions that benefit only one partner (altruistic actions, like babysitting someone else’s children so they can go on a date; Batson, 2011; Fehr & Fischbacher, 2003) and interactions that benefit both parties unequally (like one person buying two tickets to attend a concert together; Andreoni, 1990; Bekkers & Wiepking, 2011). All scenarios involved dyadic interactions in which one person actively and voluntarily performs a generous act for another, which are contexts that should evoke strong expectations of direct reciprocity (Tatone & Csibra, 2020; Widlok, 2016).

### Methods

#### Participants.

Convenience samples of adult fluent English speakers from the United States were recruited on the online crowdsourcing platform Prolific. Sample sizes were all determined in advance and preregistered (see Data and Code Availability Statements for details). Participants who completed an experiment were excluded from the subsequent experiments. Data were excluded from participants who did not pass an attention check or indicated at the end of the experiment that they did not understand the instructions. Participants gave informed consent, and all procedures were approved by the MIT Committee for the Use of Humans as Experimental Subjects.

For Study 1, we recruited 59 participants (26 female, 30 male, 3 nonbinary; ages 20–62, *M*(*SD*) age = 36.2(12.5)). For Studies 1–4, participants were paid $5 for completing an experiment, for an estimated pay of $15/hour.

#### Procedure.

**Figure 1.**
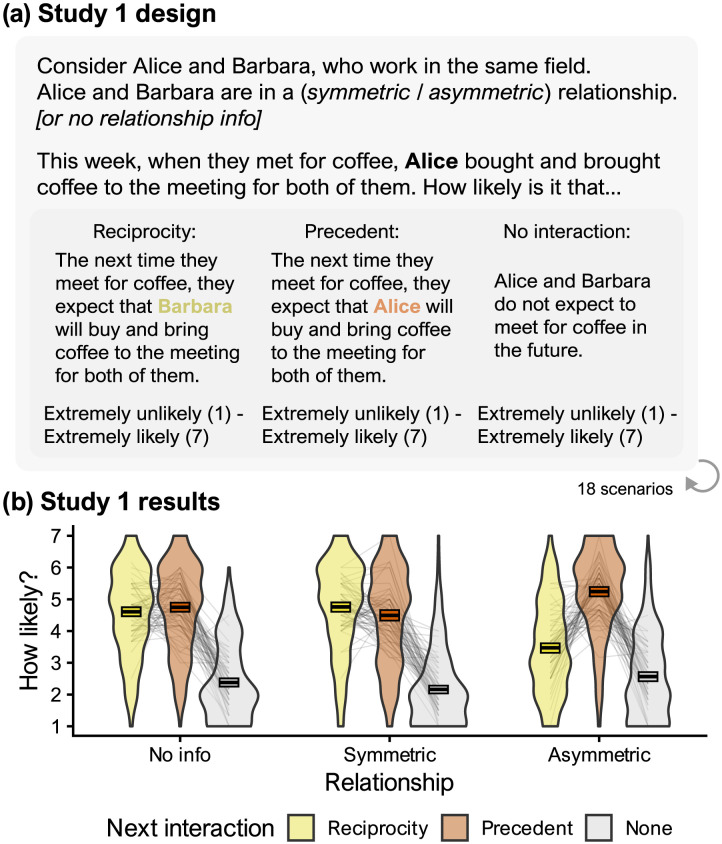
Study 1. Participants **(a)** saw information about a social relationship and one generous act, and predicted the next action. Results are shown in **(b)**. Violins show the distribution of individual trial ratings. Black boxes show bootstrapped 95% confidence intervals of the mean. Thin lines connect per-subject condition means across the three expected-action responses.

In Study 1 (Figure 1), the scenarios either omitted information about the relationship between the two characters, or described that the two characters were in an “asymmetric” relationship where one character has more power/status/influence than the other person, or a “symmetric” relationship where the two characters have equal power/status/influence. In the asymmetric condition, participants were not told which character was higher-ranked; Study 2 was designed to address this by manipulating the direction of the asymmetry. For each scenario, after one character did a generous act, participants used a 7-point Likert scale to evaluate how much the characters expected (1) the other character would do that act the next time (‘reciprocity’), (2) the same character would do the act the next time (‘precedent’), and (3) the interaction would not happen again (Figure 1a).

For all experiments, participants read each of the scenarios, and the assignment of scenarios to conditions was counterbalanced across participants. The trial sequence was randomized. The two characters interacting were always the same gender, as indicated by names and pronouns.

### Results and Discussion

To test our predictions for Studies 1–3, we used linear mixed-effects regression models to predict participants’ responses, including categorical fixed effects of relationship condition and next action (i.e., the three Likert responses) along with their interaction, and random intercepts for each scenario and participant, followed by planned contrasts between the estimated marginal means. Because these contrasts test specific, pre-specified hypotheses, *p* values are reported without adjustment for multiple comparisons^1^.

When participants were given no information about the relationship between the dyads, neither reciprocation (*M* = 4.61, *SE* = 0.09, 95% CI [4.43, 4.78]) nor following a precedent (*M* = 4.75, *SE* = 0.09, 95% CI [4.57, 4.93]) was judged to be more likely (Δ*M* = 0.14 [−0.06, 0.34], *t*(3095) = 1.39, *p* = 0.168, *d* = 0.10). People had strong expectations that generous acts lead to future social interactions: Either partner being generous a second time was judged more likely than no future interaction (Δ*M* = 2.30 [2.12, 2.47], *t*(3095) = 25.91, *p* < 0.001) (Figure 1b, left).

Minimal verbal descriptions of the structure of the relationship changed people’s expectations for who would be generous. In a relationship described as ‘symmetric’, people expected the dyad to alternate the generous role (*M* = 4.75, *SE* = 0.09, 95% CI [4.58, 4.93]) rather than follow a precedent (*M* = 4.49, *SE* = 0.09, 95% CI [4.31, 4.67]; Δ*M* = −0.26 [−0.47, −0.06], *t*(3095) = −2.58, *p* = 0.010, *d* = −0.19). In a relationship described as ‘asymmetric’, people expected the dyad to follow a precedent (*M* = 5.25, *SE* = 0.09, 95% CI [5.07, 5.43]) rather than alternate who was generous (*M* = 3.48, *SE* = 0.09, 95% CI [3.30, 3.65]; Δ*M* = 1.77 [1.57, 1.97], *t*(3095) = 17.29, *p* < 0.001, *d* = 1.30) (Figure 1b). This pattern was robust across scenarios (Supplementary Material Figure S1) and when using within-subject *z*-scored ratings or ordinal regression to address potential ceiling effects (Supplementary Material Section A.3).

Overall, observing the same action created systematic and contrasting expectations, depending on minimal abstract information about the social relationship. In symmetric equal relationships, one partner playing the generous role created the expectation that partners would alternate, generating reciprocity. In asymmetric hierarchical relationships, the same generous interaction created the opposite expectation: that the same person would be generous again.

Reciprocity was only expected over a precedent in equal relationships. The presence of a minimal cue about an asymmetry led to strong shifts in expectations, toward following a precedent. What drives this preference for precedent, and how can such minimal information so dramatically reshape expectations, even against established patterns from existing experimental studies? We address these questions in the studies that follow.

## STUDY 2: GENEROUS ACTS SET A PRECEDENT IN BOTH DIRECTIONS OF HIERARCHICAL RELATIONSHIPS

Study 1 showed that asymmetric relationships lead to expectations of precedent, but did not specify the direction of the asymmetry. In hierarchical relationships, the relative ranking of the generous actor—whether they are lower-ranked or higher-ranked—could matter. In Study 2, we tested whether the direction of the hierarchy matters in people’s expectations.

One possibility is that a generous act in a hierarchical relationship sets a precedent in both directions: whether generosity flows upward or downward in a hierarchy, the same person is expected to be generous again. Alternatively, people might expect a precedent primarily when lower-ranked individuals benefit higher-ranked individuals, given previous research suggesting that higher-ranked people are typically expected to benefit from uneven interactions (Emerson, 1964; Grueneisen & Tomasello, 2017; Le Pargneux & Cushman, 2025; Malinowski, 1922; but see Yan, 1996). We tested these possibilities in Study 2.

### Methods

#### Participants.

For Study 2, we recruited 60 participants (28 female, 27 male, 5 nonbinary; ages 18–65, *M*(*SD*) age = 33.0(10.9)), and excluded 1 participant.

#### Procedure.

We gave participants information about the *direction* of the asymmetry between the two characters interacting, specifically whether the character performing the initial generous act had more or less “power, influence and status” than the person receiving the initial act. The rest of the procedure was the same as in Study 1.

#### Scenario Validation Experiment.

Generous behavior has been defined as an act where one person incurs a cost, to benefit another (Fehr & Fischbacher, 2003), and these measures have been shown to affect adult and children’s evaluations of generosity (Radovanovic et al., 2023; Sommerville et al., 2018). Thus, to test whether there is separable variance in the scenarios that could explain variance in the actual experiments, we also ran two experiments measuring the relative cost and benefit (*N* = 58 for benefit, *N* = 59 for effort, Supplementary Material Figure S3) between the two people interacting in each scenario.

In each experiment, we showed participants all 18 scenarios, each scenario displaying one generous action that one character performs for the other. In the ‘benefit’ experiment, we asked participants how much each of the two characters in the scenario benefits from the interaction, compared to if they don’t interact. In the ‘effort’ experiment, we asked participants how much effort each character puts into the interaction, compared to if they don’t interact. Thus, participants answered two questions on each trial, corresponding to the two characters in the scenario, each using a 7 point Likert scale (‘not at all’ (1)–‘extremely’ (7)).

The scenarios in Supplementary Material Figure S3 are ordered based on the differential benefit between the two people interacting. In general, people expect the recipient of the generosity to benefit more than the generous actor, and that the generous actor puts in more effort than the recipient (Supplementary Material Figure S3a). Additionally, relative benefit generally corresponds to relative effort (scenarios where the generous actor puts in relatively more effort also correspond to the scenarios where they benefit less) (Supplementary Material Figure S3a). Some scenarios are more uneven in either benefit or effort: For example, in the ‘restaurant’ scenario, participants expect that the generous actor (who offers to go to the recipient’s preferred restaurant) puts in comparatively less effort, compared to how much the recipient benefits from the interaction.

Overall, the results of these experiments suggest that, in general, our scenarios display uneven benefit or uneven effort (one character puts in more effort, or benefits more, than the other), and that they cover a wide range of benefit and effort.

### Results and Discussion

**Figure 2.**
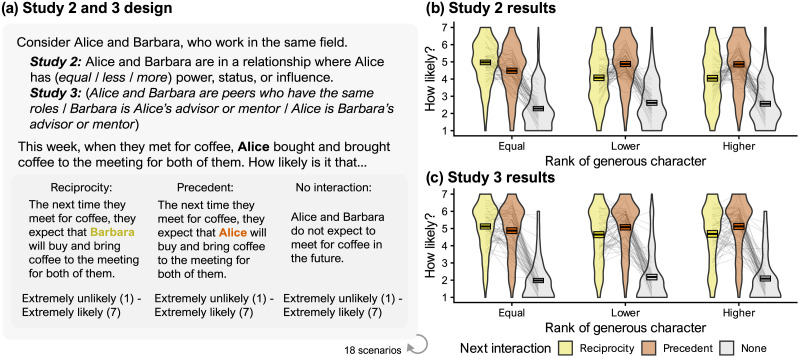
Study 2 and 3. Participants **(a)** saw information about a social relationship and one generous act, and predicted the next action. In Study 2, participants were shown information about the relative power, status, or influence between the two characters. In Study 3, participants were shown a description of a concrete relationship. Results for Study 2 are shown in **(b)** and results for Study 3 are shown in **(c)**. Violins show the distribution of individual trial ratings. Black boxes show bootstrapped 95% confidence intervals of the mean. Thin lines connect per-subject condition means across the three expected-action responses.

The results from Study 1 replicated: When the relationship was hierarchical, participants expected generous acts to set a precedent (*M* = 4.86, *SE* = 0.08, 95% CI [4.71, 5.02]) rather than elicit reciprocity (*M* = 4.06, *SE* = 0.08, 95% CI [3.90, 4.22]; Δ*M* = −0.80 [−0.95, −0.66], *t*(3090) = −11.04, *p* < 0.001, *d* = −0.59). Participants had the opposite expectation in equal relationships, expecting reciprocity (*M* = 4.98, *SE* = 0.09, 95% CI [4.79, 5.16]) over a precedent (*M* = 4.48, *SE* = 0.09, 95% CI [4.30, 4.67]; Δ*M* = 0.49 [0.29, 0.69], *t*(3090) = 4.76, *p* < 0.001, *d* = 0.36) (Figure 2b).

Participants were equally likely to expect a precedent from lower-ranked (*M* = 4.86, *SE* = 0.09, 95% CI [4.67, 5.04]) and higher-ranked (*M* = 4.87, *SE* = 0.09, 95% CI [4.69, 5.06]) characters (Δ*M* = 0.02 [−0.19, 0.22], *t*(3098) = 0.16, *p* = 0.871, *d* = 0.01; *BF*_01_ = 11.79, indicating strong evidence for the null) (Figure 2b). The expectations of a precedent held in both cases.

There was variation in people’s responses across scenarios (Supplementary Material Figure S2). As an exploratory analysis, we measured the influence of the degree of asymmetry of costs and benefits incurred by the specific kind of generous interaction in the scenario, on participants’ expectations. For each direction of hierarchical relationship, we conducted two separate linear mixed-effects regressions predicting how much people expected a precedent (probability of choosing a precedent vs. reciprocity, computed from the Likert responses) from differential benefit and from differential effort, as measured in the scenario validation experiment. Costs and benefits varied across scenarios (Supplementary Material Figure S3a) but had very little influence on expectations of reciprocity versus precedent setting. When the lower-ranked character played the generous role, the relative costs or benefits of the action did not affect how much people expected a precedent (effort *b* = 0.00, *t*(16.04) = 0.21, *p* = 0.835; benefit *b* = −0.03, *t*(16.01) = −1.47, *p* = 0.162) (Supplementary Material Figure S4). When the higher-ranked character was generous, cost did not influence expectations (*b* = 0.02, *t*(16.03) = 0.58, *p* = 0.567) (Supplementary Material Figure S4b), although for larger differential benefits, participants did consider following a precedent by the higher-ranked character slightly less likely (*b* = −0.06, *t*(15.94) = −2.50, *p* = 0.024) (Supplementary Material Figure S4a).

In sum, replicating Study 1, participants expected generous acts to elicit reciprocity in equal relationships, but to set a precedent in hierarchical relationships. Study 2 revealed that these results generalize across both directions of a hierarchical relationship. Generous acts set a precedent regardless of whether the generous person is lower- or higher-ranked.

## STUDY 3: CONCRETE LABELS FOR RELATIONSHIPS GENERATE THE SAME SYSTEMATIC EXPECTATIONS AS ABSTRACT LABELS

Archetypal symmetric and asymmetric social relationships are remarkably consistent across a large range of cultures and time periods of human history (Cheng et al., 2025). Studies 1 and 2 provided only minimal abstract descriptions of relationships. Study 3 (Figure 2a and c) aimed to test whether people’s vernacular intuitions about specific familiar roles map onto the more abstract distinction between symmetric and asymmetric relationships used in the previous two studies.

### Methods

#### Participants.

For Study 3, we recruited 60 participants (28 female, 32 male, 0 nonbinary; ages 18–79, *M*(*SD*) age = 40.4(15.2)), and excluded 3 participants.

#### Procedure.

The vignettes in Study 3 contained concrete labels for archetypal symmetric and asymmetric relationships (e.g., coworkers vs. advisor and advisee, cousins vs. uncle and nephew; see Figure 2a, and Supplementary Material Table S3 for a list of the specific relationships). The procedure was otherwise the same as for Study 2.

### Results and Discussion

The results replicated Studies 1 and 2 (Figure 2c). In equal relationships, participants expected reciprocity (*M* = 5.13, *SE* = 0.11, 95% CI [4.91, 5.34]) over precedent (*M* = 4.88, *SE* = 0.11, 95% CI [4.66, 5.10]) (Δ*M* = 0.25 [0.03, 0.46], *t*(2982) = 2.29, *p* = 0.022, *d* = 0.18). In hierarchical relationships, people expected precedent (*M* = 5.10, *SE* = 0.10, 95% CI [4.91, 5.29]) over reciprocity (*M* = 4.67, *SE* = 0.10, 95% CI [4.47, 4.86]) (Δ*M* = −0.43 [−0.58, −0.29], *t*(2982) = −5.70, *p* < 0.001, *d* = −0.31). The relative direction of the hierarchical relationship did not alter these expectations: There was no difference in how much participants expected a precedent, when the generous character was lower-ranked (*M* = 5.10, *SE* = 0.11, 95% CI [4.88, 5.32]) versus higher-ranked (*M* = 5.11, *SE* = 0.11, 95% CI [4.89, 5.33]) (Δ*M* = −0.01 [−0.22, 0.20], *t*(2991) = −0.10, *p* = 0.922, *d* = −0.01; *BF*_01_ = 11.10, indicating strong evidence for the null) (see Supplementary Material Figure S5 for the scenario-specific responses). Neither differential cost nor benefit of the interaction influenced expectations when the lower-ranked character was generous (cost: *b* = −0.00, *t*(16.78) = −0.22, *p* = 0.831, benefit: *b* = −0.02, *t*(16.44) = −1.11, *p* = 0.281) (Supplementary Material Figure S4c and d). When the higher-ranked character was generous, differential cost did not influence expectations, but, as in Study 2, high ranked people were judged slightly less likely to repeat generous acts with large differential benefits (cost: *b* = −0.01, *t*(16.61) = −0.49, *p* = 0.633; benefit: *b* = −0.05, *t*(15.89) = −3.21, *p* = 0.006) (Supplementary Material Figure S4c and d).

## STUDY 4: EXPECTATIONS FOR WHO WILL BE GENEROUS ARE BASED ON THE SPECIFIC INTERACTION AND THE GENERAL PRECEDENT

Studies 2 and 3 showed that people overall expected a generous act to set a precedent in hierarchical relationships, regardless of whether the generous actor was lower- or higher-ranked. However, expectations for who would be generous also varied across the specific interaction scenarios (Supplementary Material Figures S2 and S5) in a way that could not be explained by relative cost or benefit. In Study 4, we investigate another plausible source of the variation: people’s prior expectations for specific types of interactions in hierarchical relationships.

**Figure 3.**
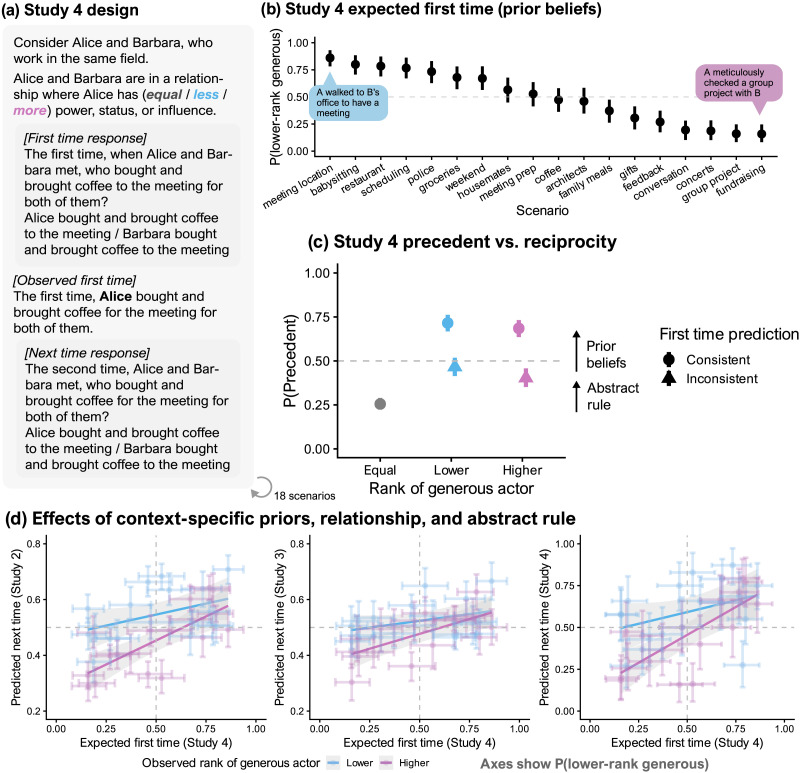
Study 4. Participants **(a)** reported their priors for who would be generous, and after observing who was actually generous (assigned by the experiment), predicted who would be generous the next time. Participants’ **(b)** priors cover a range of expectations. **(c)** shows aggregate expectations that the next interaction will follow the precedent set by the first interaction (*y*-axis), based on whether the observed first interaction was consistent (circle) or inconsistent (triangle) with the participant’s prior beliefs. In hierarchical relationships, **(d)** shows how people’s prior expectations for each scenario (*x*-axis; as shown in (b)) and the experimentally manipulated observations of the first interaction (color) predict who they expect to be generous in the second interaction (*y*-axis). Each point corresponds to one scenario. Error bars are bootstrapped 95% confidence intervals.

Specific scenarios could evoke different scripts for the roles different types of people should play (Jara-Ettinger & Dunham, 2025). Given these scripts, people might have prior expectations for who should be generous, even without observing any social interactions (implicit coordination; Schelling, 1980). For example, in many US contexts, it is expected that the lower-ranked person walks to the higher-ranked person’s office to have a meeting, whereas the higher-ranked person is expected to conduct detailed checks on important tasks. These priors could be local and arbitrary, but might also reflect generalizable associations with different kinds of relationships (Nisbett & Miyamoto, 2005). If these scenario-based priors are strong, then people might expect roles to repeat just because those roles are likely in each interaction taken separately. This mechanism could explain scenario-specific variance in Study 2 and 3: Observing an action that aligns with one’s prior beliefs could lead to strong expectations that the same action will happen again. In Study 4 (Figure 3), we explicitly measure these scenario-specific priors and assess their influence on people’s expectations for when others will follow precedents.

In addition to scenario-specific priors, people could also be applying an abstract rule that people should repeat their previous roles in coordinated actions. If an abstract rule governs expectations of precedent-following, then people would predict repeated generous roles based on observed behavior alone. For example, if a specific higher-ranked person was observed walking to the lower-ranked partner’s office for a meeting, contrary to the typical cultural script, then on the next interaction they would be expected to repeat their previous role and walk to the lower-ranked partner’s office again.

### Methods

#### Participants.

For Study 4, we recruited 120 participants (59 female, 60 male, 1 nonbinary; ages 18–85, *M*(*SD*) age = 37.8(12.0)), and excluded 7 participants.

#### Procedure.

In Study 4, participants were given minimal information about the relative rank of the two people interacting, as in Study 2, and then we explicitly dissociated the contributions of scenario-specific priors and an abstract rule by measuring expectations at two time-points. First, participants predicted who would be generous (two-alternative forced choice) based solely on the vignette, reflecting their prior beliefs. These beliefs should be probabilistic and open to revision. Then, after observing who was actually generous in the experimental manipulation (either confirming or violating their prior beliefs), participants predicted who would be generous in the next interaction (Figure 3a).

### Results and Discussion

Participants’ prior beliefs^2^ are shown in Figure 3b. Unlike abstract transfers of money or points, the vignettes evoke a wide range of expectations for which character in a hierarchical relationship would be generous in their first interaction. In some scenarios, participants expected the lower-ranked character to act generously, and in some scenarios, participants expected the higher-ranked character to act generously. This variation was not predicted by the differential costs or benefits in each scenario (two separate logistic mixed-effects regressions predicting whether the lower- or the higher-ranked character was initially generous, with random intercepts for scenario and participant; from relative benefit *b* = 0.38, *z* = 1.50, *p* = 0.134, OR = 1.47 [0.89, 2.42]; and from relative effort *b* = −0.11, *z* = −0.43, *p* = 0.667, OR = 0.89 [0.53, 1.50]) (Supplementary Material Figure S4e).

We then examined how expectations shifted after observing a social interaction between the two characters. We ran a logistic mixed-effects regression predicting the expected sequence of actions (‘reciprocity’ if the predicted next action was the opposite of the observed first action; ‘precedent’ if the predicted next action was the same as the observed first action) from the observed first action (‘generous lower-rank’ vs. ‘generous higher-rank’) and whether the observation was consistent with expectations, along with their interaction; including random intercepts for scenario and participant. In equal relationships, observing one person act generously led to expectations of reciprocity—that the opposite partner would be generous next (*M* = −1.41, *SE* = 0.22, 95% CI [−1.84, −0.98]). By contrast, in hierarchical relationships, people had stronger expectations that the observed partner would act generously again, following a precedent; although the size of the effect depended on whether the observed first interaction matched or violated participants’ prior beliefs (Figure 3c). When the observed action aligned with participants’ prior beliefs (i.e., when both the persistence of prior beliefs and an abstract rule could contribute to precedent-following), people were very likely to expect that the action would repeat, regardless of whether the generous actor was higher- or lower-ranked (lower: *M* = 1.16, *SE* = 0.24, 95% CI [0.70, 1.63]; higher: *M* = 0.87, *SE* = 0.24, 95% CI [0.40, 1.33]; *BF*_01_ = 2.46, indicating anecdotal evidence for the null) (both *p* < 0.001). Even when the observed action contradicted participants’ prior beliefs (i.e., when only an abstract rule could contribute to precedent-following), participants still expected the observed actor to be generous again, more than in equal relationships (lower: *M* = −0.20, *SE* = 0.23, 95% CI [−0.66, 0.26]; higher: *M* = −0.39, *SE* = 0.23, 95% CI [−0.85, 0.07]) (both *p* < 0.001). In other words, given a minimal description of an asymmetric relationship, participants were less likely to expect reciprocity and more likely to expect a precedent—even when observed interactions violated participants’ prior beliefs. Thus both scenario-specific priors and an abstract rule, activated based on knowledge of a hierarchical social relationship, contribute independently to expectations of precedent-following.

We examined how this behavioral pattern was shaped by expectations for the first action, across vignettes. We ran a logistic mixed-effects regression predicting ‘expected next time’ responses from ‘expected first time’ and ‘observed first time’, along with their interaction, and random intercepts for each scenario and participant. Across individual vignettes (Figure 3d, right), observers’ predictions for the second interaction were additively shaped by both their scenario-specific prior beliefs (*x*-axis) and their experimentally manipulated observations of the first interaction (color): there were strong main effects of both the expected actor (*b* = 0.51, *z* = 8.34, *p* < 0.001, OR = 1.66 [1.47, 1.87]) and the observed actor (*b* = 0.29, *z* = 5.14, *p* < 0.001, OR = 1.34 [1.20, 1.50]) from the first interaction, on expectations for who would be generous in the next interaction (*y*-axis).

One concern might be that participants’ prior beliefs influenced expectations for the second interaction only because the experiment required participants to explicitly report these priors. To check this possibility, we conducted exploratory analyses using data from Studies 2 and 3. The regression model followed the same structure, but the response variable was instead relative probabilities computed from the Likert responses (specifically, the probability of the lower-ranked character acting generous next). All of the results replicated (Study 2: ‘expected first time’ *b* = 0.25, *t*(16.18) = 9.46, *p* < 0.001; ‘observed first time’ *b* = 0.09, *t*(685.45) = 7.54, *p* < 0.001; Figure 3d, left) (Study 3: ‘expected first time’ *b* = 0.15, *t*(16.51) = 3.84, *p* = 0.001; ‘observed first time’ *b* = 0.05, *t*(663.17) = 4.21, *p* < 0.001; Figure 3d, middle). Additional variance in Study 3 was explained by including features of the concrete relationship labels derived from a large-scale study (see Supplementary Material Section A.7 for detail).

In sum, Study 4 replicated the key findings of the earlier studies, and explained scenario specific variance by dissociating the effect of scenario-specific prior beliefs. Predictions of precedent-following in hierarchical relationships are shaped by both these priors and an abstract rule that what happened last time should happen again. In everyday life, these inferences are difficult to separate because culturally specific scripts reflect what usually happens. Naturalistic measurements of social experience would rarely capture interactions in which expected social roles are violated. By experimentally dissociating prior beliefs from experimentally manipulated observations, we showed that people rely on both. Participants’ prior expectations for who would be generous were strengthened when aligned with observed actions, and weakened when they were violated. In both cases, however, people activated an abstract rule that previous roles in an interaction should repeat in hierarchical relationships. A minimal cue to hierarchy was sufficient to trigger these expectations.

## STUDIES 5–6: COSTLY FIRST PERSON CHOICES REFLECT THE SAME EXPECTATIONS

**Figure 4.**
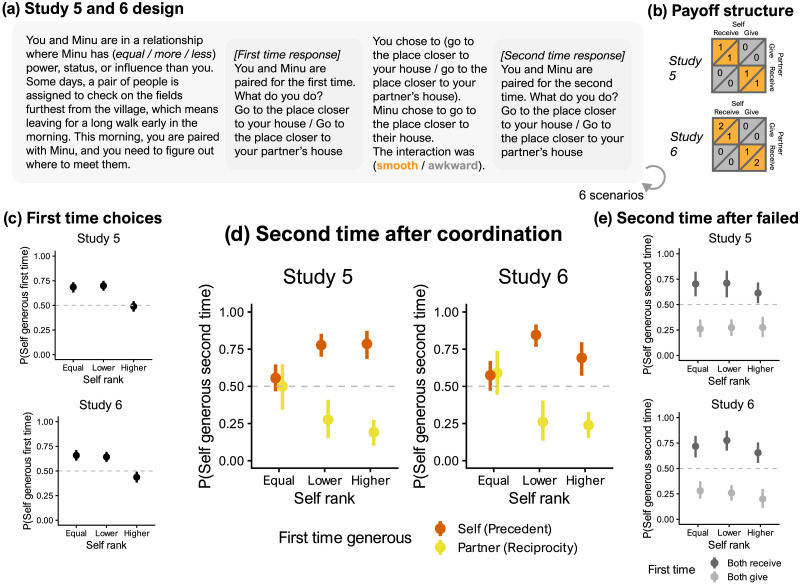
Studies 5 and 6. Participants **(a)** chose what they would do the first time and the second time they interact with their partner. The explicit incentives in the experiments are shown in **(b)**: Participants were incentivized based on successfully coordinating and choosing complementary actions in both studies, but accepted a personal cost for being generous in Study 6. Participants **(c)** were generally generous in their first choices, except for when they were higher ranked. Following a successful coordination, participants **(d)** repeated their actions if they were lower or higher ranked, but switched their actions if they were in an equal relationship. Following an unsuccessful coordination, they **(e)** chose the complementary action to what their partner chose the first time. Error bars are bootstrapped 95% confidence intervals.

Studies 1–4 measured third-party observers’ predictions of who will be generous, using vignettes reflecting middle-class U.S. contexts. In Studies 5 and 6, we tested whether these expectations also shape people’s own choices in incentivized games. Incentivized coordination and bargaining games are the paradigm that has produced some of the strongest evidence for reciprocity and turn-taking as default strategies (Camerer, 2003; McKelvey & Palfrey, 2001). The predominance of turn-taking in these games may itself reflect the fact that they are typically played between strangers, who are implicitly equal in status (Hoffman et al., 1994). If minimal relationship cues can shift behavior even in this setting—where there are explicit financial stakes and minimal culturally specific priors—it would provide a strong test of whether the expectations documented in Studies 1–4 generalize beyond hypothetical vignette judgments. In sum, Studies 5 and 6 (Figure 4) test whether minimal relational information is sufficient to alter behavior even in a setting designed to maximize strategic thinking.

### Methods

#### Participants.

For Study 5, we recruited 156 participants (79 female, 76 male, 1 nonbinary; ages 19–77, *M*(*SD*) age = 39.2(11.8)) and excluded 3 participants. For Study 6, we recruited 162 participants (76 female, 84 male, 2 nonbinary; ages 19–78, *M*(*SD*) age = 40.7(13.7)) and excluded 4 participants. There were two attention checks and participants were excluded if they missed both attention checks. In each of Studies 5 and 6, participants received $3 for completing the experiment, with a bonus of up to $3 based on how many points they earned in the task, for an estimated pay of $15/hour.

#### Procedure.

Participants were told to imagine that they were visiting a remote society with unknown norms (to minimize the effect of participants’ culturally specific priors), where they would interact with multiple individuals who understood these norms (Figure 4a). Participants interacted twice each with simulated partners described as having either more, equal, or less power/status/influence, across six scenarios involving asymmetric interactions with one generous role (e.g., serving food first or carrying heavier objects; see Supplementary Material Table S4 for all scenarios). Participants chose a first role (two-alternative forced choice between giving and receiving generosity) and then received immediate feedback of their partner’s choice and whether the coordination was “smooth” (complementary choices, which are required for successful coordination in these role-differentiated scenarios) or “awkward” (non-complementary choices). Our key hypotheses concerned what participants chose to do during the second interaction, based on the outcome of the first interaction and the minimal description of the relationship.

Each of the six scenarios appeared in one of the six conditions (partner rank: ‘equal’ vs. ‘lower’ vs. ‘higher’; partner action: ‘give’ vs. ‘receive’). Like in the previous experiments, the assignment of conditions to scenarios was balanced within and across participants. The trial sequence was randomized, with the constraint that the ‘first time’ interaction for a certain partner always happens before the ‘second time’ interaction with that partner.

The two studies differed only in participant incentives (Figure 4b). In Study 5, participants received one point for smooth interactions (coordination: the partners chose complementary roles) and no points for awkward interactions (miscoordination: partners chose the same role). Although the financial payoffs for giving and receiving were identical in Study 5, the vignettes described one role as performing a generous act and the other as receiving it, preserving the narrative cost asymmetry of the vignette studies. Study 5 thus serves as an intermediate between the vignette paradigm of Studies 1–4 and a fully incentivized paradigm. In Study 6, the incentives corresponded to a Leader-Follower game (archaically named Battle of the Sexes; Luce & Raiffa, 1957): two points for smoothly receiving a generous act, one point for smoothly doing a generous act, and no points for awkward interactions. Thus, in Study 6, participants incurred a direct financial cost when choosing to act generously compared to receiving generosity.

### Results and Discussion

#### People Expect Others to Follow a Precedent, Except After Successful Interactions in Equal Relationships.

In these games, participants expressed predictions for others’ actions behaviorally, by making complementary choices. To test our predictions, we ran a logistic mixed-effects regression predicting participants’ likelihood of expecting a precedent (versus reciprocity), from the social relationship and whether the first interaction was smooth or awkward, along with their interaction; with random intercepts for scenario and participant; followed by post-hoc contrasts between the marginal means. Consistent with our key hypothesis, participants’ choices for the second social interaction varied depending on the minimal description of their relationship with the partner (Figure 4d). After a smooth first interaction in a hierarchical relationship, participants continued choosing the complement of their partner’s first choice. That is, participants expected to coordinate with both lower-ranked and higher-ranked partners by following a precedent. This was the case even in Study 6, where participants followed a precedent even when they were in the disadvantaged role (i.e., repeatedly giving at a personal cost) (Study 5 lower: *M* = 1.60, *SE* = 0.24, 95% CI [1.12, 2.07]; higher: *M* = 1.48, *SE* = 0.22, 95% CI [1.04, 1.91]) (Study 6 lower: *M* = 1.18, *SE* = 0.20, 95% CI [0.78, 1.57]; higher: *M* = 1.65, *SE* = 0.24, 95% CI [1.18, 2.13]). By contrast, after a smooth first interaction in an equal relationship, participants were comparatively more likely to reciprocate their partner’s initial action (all *p* < 0.002), accepting the risk of miscoordination in service of balanced exchange. This pattern was robust across both Studies 5 (symmetric payoffs) (*M* = 0.23, *SE* = 0.20, 95% CI [−0.17, 0.62]) and 6 (asymmetric payoffs) (*M* = 0.14, *SE* = 0.19, 95% CI [−0.24, 0.51]). Even in Study 5, where the financial payoffs were identical for both roles, participants treated the narrative distinction between giving and receiving as meaningful—sacrificing coordination efficiency for turn-taking, but only when the relationship was described as equal.

If the first interaction resulted in miscoordination, participants chose the opposite of what they and their partner initially chose, giving if they had chosen to receive and receiving if they had chosen to give, in all three relationships (Figure 4e).

#### What Contributed to People’s Choices to be Generous?

For the first interaction (Figure 4c), participants chose to initiate generosity when they were equal-ranked and when they were lower-ranked (potentially because of reputational or status-related benefits; Flynn & Yu, 2021), but not when they were higher-ranked. This was the case even in Study 6, when there were explicit financial disincentives for choosing to act generously.

For the second interaction, several factors could influence participants’ choices in addition to relationship structure. People might rely on habits and simply repeat their own previous actions (Wood & Rünger, 2016). People might overall feel motivated to reciprocate generous acts they received (Fehr & Gächter, 2000; Gouldner, 1960). People might apply a simple learning rule to repeat their action after successful coordination and change actions after unsuccessful coordination (the win-stay lose-shift strategy; Homans, 1958; Nowak & Sigmund, 1993). Finally, each person could have an overall baseline motive to act prosocially and benefit others (Aknin et al., 2018; Rand & Nowak, 2013).

We predicted participants’ choice to be generous in the second interaction, based on all of the factors above, plus an additional interaction term between the partner’s first choice and the social relationship, with random intercepts for scenario and participant. An effect of the participant’s first choice corresponds to habit (whether participants are likely to give when they chose to give the last time), an effect of the partner’s first choice corresponds to straightforward reciprocity (whether participants are more likely to give when their partner chose to give the last time), an interaction effect corresponds to an effect of a ‘win-stay lose-shift strategy’ (complementary choices leading to a smooth interaction; the levels are effect-coded, so the negative of the interaction effect directly corresponds to WSLS), and an intercept corresponds to baseline generosity.

This full model explained the results better than a simpler model that did not include the social relationship (likelihood-ratio test; Study 5: *χ*^2^(4) = 11.68, *p* = 0.020; Study 6: *χ*^2^(4) = 26.64, *p* < 0.001). The interaction term was significant (Type III ANOVA; Study 5: *χ*^2^(2) = 9.38, *p* = 0.009; Study 6: *χ*^2^(2) = 17.98, *p* < 0.001). The only other predictor across the two experiments was a negative effect of simple reciprocity (Study 5: *b* = −0.86, *z* = −11.39, *p* < 0.001, OR = 0.42 [0.37, 0.49]; Study 6: *b* = −0.91, *z* = −12.20, *p* < 0.001, OR = 0.40 [0.35, 0.47]): With the exception of a successful first interaction in an equal relationship, participants generally chose the opposite of their partner’s first choice whether the first interaction was successful or not, indicating that participants expected their partner to perform the same actions again, following a precedent. There was a small negative effect of habit in Study 6 (*b* = −0.16, *z* = −2.06, *p* = 0.039, OR = 0.86 [0.74, 0.99]) but not in Study 5 (*b* = −0.01, *z* = −0.17, *p* = 0.863, OR = 0.99 [0.85, 1.15]). There was no evidence of a heuristic rule like win-stay-lose-switch (Study 5: *b* = −0.09, *z* = −1.18, *p* = 0.237, OR = 0.91 [0.79, 1.06]; Study 6: *b* = −0.12, *z* = −1.61, *p* = 0.107, OR = 0.89 [0.76, 1.03]) or baseline generosity in the second interaction (Study 5: *b* = −0.07, *z* = −0.97, *p* = 0.330, OR = 0.93 [0.80, 1.08]; Study 6: *b* = 0.01, *z* = 0.15, *p* = 0.882, OR = 1.01 [0.86, 1.18]).

In sum, participants’ own financially incentivized behaviors in repeated asymmetric coordination games reflect their prediction that partners are likely to follow a precedent across interactions. Participants were less likely to expect a precedent and more likely to expect reciprocity, only in equal relationships after a successful first social interaction. As in Studies 1–4, minimal context about relationships and daily-life social interactions was sufficient to induce these expectations.

## GENERAL DISCUSSION

We found that people form distinct and systematic expectations for sequences of generous acts. In most situations, a single generous act established a precedent: if the context arose again, the same person was expected to be generous in the same way again. Yet people’s predictions and behavior were substantially altered by minimal descriptions of social relationships. In asymmetric hierarchical relationships, following a precedent became more likely. Expectations of reciprocity were restricted to equal relationships. We replicated this pattern six times, across third-party hypothetical judgments and first-person coordination games with financial payoffs. Precedents were anticipated in the absence of any relationship information (Study 1), in asymmetric relationships (Studies 1–6), both up and down a hierarchy of power or status (Studies 2–6), in concrete familiar asymmetric relationships like uncle-nephew or boss-employee (Study 3), and across a range of specific vignettes (Study 4) and action costs and benefits (Studies 2–4). These expectations influenced people’s own choices (Study 5), even when following a precedent meant repeatedly being generous and thus repeatedly receiving a lower payoff than the partner (Study 6).

Our results contrast with many studies of lab-based coordination games between strangers, in which players typically expect direct reciprocity, and use partner choice to avoid partners who try to repeatedly benefit (Barclay, 2016; Lau & Mui, 2008; McKelvey & Palfrey, 2001). The predominance of reciprocity in these paradigms may reflect a default assumption of equal rank among strangers (Hoffman et al., 1994; Tatone & Csibra, 2020). By contrast, our results suggest that this assumption is brittle, and can be disrupted by even minimal social context and framing (DeScioli & Krishna, 2013; Hoffman et al., 1994). Compared to standard paradigms, we made two key methodological changes: our vignettes described meaningful everyday asymmetric social interactions (giving gifts, household labor, carrying a heavy object) rather than simple exchanges of money; and we situated the social interactions in minimal but meaningful structured social relationships. These changes led to systematic shifts in expectations. The preference for reciprocity in equal relationships was also consistently smaller in magnitude than the strong shift toward precedent in hierarchical relationships, suggesting that reciprocity among equals may be a weaker baseline expectation rather than the robust default norm assumed by much prior work.

This research directly tests the prediction, articulated most explicitly by Graeber (2011) and grounded in relational theories (Clark et al., 2017; Fiske, 1992), that expectations for sequences of generosity are context-sensitive expectations governed by the perceived structure of social relations. This view challenges one way economists traditionally conceptualize human behavior. Classic economic accounts are grounded in the assumption that human social and moral life is motivated by a sense of justice in the form of reciprocity and fairness (Homans, 1958; Lévi-Strauss, 1952). According to this view, the psychological principle that actions and debts should be reciprocated, is not only used as a descriptive theory of social behavior but also as a normative justification for large-scale market-based economies, where the payoffs of actions are explicitly quantified and meant to be balanced (Fehr & Gächter, 2000; Sugden, 2005). By contrast, Graeber draws on extensive ethnographic evidence describing everyday generous acts, to argue that people only have these expectations in social relationships that are actually or potentially between social equals. This theoretical claim is ultimately a psychological one, yet it has not been directly tested. In this paper, we addressed this gap by empirically testing the minimal conditions that evoke these context-dependent expectations.

Our research has limitations. Notably, our experiments recruited native English speakers from the United States—the kind of population typically sampled for existing experimental work demonstrating expected norms of reciprocity—to test whether minimal cues about structured social relationships could evoke contrasting expectations similar to those described in ethnographic studies. Even for this population from a society with high mobility, loose norms (Gelfand et al., 2011), and high market integration (Henrich, Ensminger, et al., 2010), minimal relationship information shifted expectations. Generalizing the current claims beyond this cultural setting would require cross-cultural research (Henrich, Heine, & Norenzayan, 2010; Rad et al., 2018). We speculate that scenario-specific cultural norms vary across cultures and historical contexts, and yet that the principle—linking a hierarchical relationship to an abstract rule that past actions should repeat, and an equal relationship to reciprocity—may be shared across societies.

Our studies focused on direct, short-term reciprocity in dyadic interactions involving active, voluntary giving—in Fiske (1992)’s framework, “equality matching” (balanced turn-taking) and “market pricing” (quantified exchange). We did not probe longer-term or indirect forms of reciprocity (Bekkers & Wiepking, 2011; Fiske, 1992; Nowak & Sigmund, 2005), communal or need-based giving where benefits are provided without bookkeeping (Clark & Mills, 2012; Hruschka, 2010)—what anthropologists call “promiscuous sharing,” characteristic of high-intimacy relationships (Fiske, 1992)—or passive transfers such as taking or leaving goods for others, which may evoke weaker reciprocal obligations (Tatone & Csibra, 2020; Widlok, 2016). None of the relationships in our studies were at the extreme ends of intimacy (e.g., spouses, parent-infant), and our response options asked about the next specific interaction rather than longer-term patterns of balance. How closeness, transfer mode, and timescale interact with hierarchy to shape exchange norms are important questions for future work.

There are at least two cognitive mechanisms that could contribute to people’s strong expectations of precedents in our experiments. First, acting predictably, as in following a precedent, is a way to simplify social coordination, increasing collective benefits. All else being equal, people expect each other to choose the same action if the same context arises again, and prefer others who are predictable in their social actions (Theriault et al., 2021). Precedents and conventions exert a powerful force, stabilizing expectations across many types of social interactions (Hawkins et al., 2019; Henrich, 2016; Lewis, 1969; Millikan, 2005). In iterated asymmetric coordination games in particular, repeating the same roles is a good strategy because each player’s policy is simple, reducing demands on memory and risks of miscoordination (Hammerstein, 2003; Stevens & Hauser, 2004). Indeed, following an asymmetric precedent is often in every individual’s own strategic best interest, including those who are repeatedly disadvantaged by the precedent and even though it sustains and entrenches unequal payoffs (O’Connor, 2019). Thus people follow precedents when the probability and costs of miscoordination are high (Prisbrey, 1993; Yu & Thompson, 2024).

Second, in our studies, expectations were influenced by described and implied social relationships (Thomas, 2026). People may expect precedent particularly in hierarchical relationships because each asymmetric interaction affirms and reinforces the overall social order (Rai & Fiske, 2011)—when hierarchy is mutually recognized as legitimate, the unequal payoffs of following a precedent may be a feature rather than a bug. A notable feature of our results is that expectations of precedent held in both directions of a hierarchical relationship: whether the generous actor was lower-ranked or higher-ranked, participants expected the same person to be generous again. The most parsimonious interpretation is that status asymmetry itself activates a precedent norm, irrespective of directionality. One might speculate that upward generosity (lower-ranked benefiting higher-ranked) signals dominance, while downward generosity signals prestige (Cheng et al., 2013; Henrich & Gil-White, 2001), and that both lead to precedent through different mechanisms. However, our data do not distinguish these possibilities, and the mapping between directionality and hierarchy type may not be straightforward, since upward voluntary giving (e.g., tributary gifts) can also occur in prestige-based or culturally legitimate hierarchies (Fiske, 1992; Henrich & Gil-White, 2001; Mauss, 1925; Yan, 1996). Whether people represent these as a single mapping (hierarchy → precedent; Graeber, 2011) or two distinct ones (dominance → lower gives, prestige → higher gives; Henrich & Gil-White, 2001) is an open question for future work.

In summary, we studied people’s social cognitive expectations of repeated sequences of generous acts. We found experimental evidence that expectations of direct reciprocity emerge specifically in the context of perceived or desired equality between individuals. In other common social situations, including interactions between people in hierarchical relationships, one generous act created the opposite expectation, that the same person would be generous in the same way again.

## ACKNOWLEDGMENTS

We thank Jae-Young Son, Kartik Chandra, Ashley Thomas, Paul Bloom, and the anonymous reviewers for helpful comments on earlier versions of this paper. Studies 1 and 2 are based on nonarchival work presented at the 45th and 46th Conferences of the Cognitive Science Society (Chen & Saxe, 2023, 2024).

## FUNDING INFORMATION

This research was supported by the Patrick J. McGovern Foundation.

## AUTHOR CONTRIBUTIONS

A.M.C.: Conceptualization; Data curation; Formal analysis; Investigation; Methodology; Software; Validation; Visualization; Writing – original draft; Writing – review & editing. R.S.: Conceptualization; Funding acquisition; Project administration; Resources; Supervision; Writing – original draft; Writing – review & editing.

## DATA AND CODE AVAILABILITY STATEMENTS

All data and code for running the experiments and reproducing the analyses and plots are available on GitHub at https://github.com/aliciamchen/generosity-actions and also deposited on Zenodo at https://doi.org/10.5281/zenodo.17477355. The experiments and analyses were all preregistered unless otherwise stated. Study 1 was preregistered (https://osf.io/fxwbh/) and collected on January 30, 2023. Study 2 was preregistered (https://osf.io/jvnca/) and collected on April 12, 2023. Study 3 was preregistered (https://osf.io/vt4kp) on April 30, 2025 and collected on April 30 and May 1, 2025. Study 4 was preregistered (https://osf.io/dfv24/) and collected on August 5, 2023. Study 5 was preregistered (https://osf.io/ag9v2/) and collected on May 16, 2025. Study 6 was preregistered (https://osf.io/zn6wh/) and collected on May 17, 2025. Note that the numbering of the studies was changed from as stated in the preregistration titles. There were minor deviations from the preregistrations, reported in the Supplementary Material Section A.1. AI tools were used to assist with code development and editing the paper. All AI-assisted output was reviewed, edited, and verified by the authors, who take full responsibility for the final work.

## Notes

^1^ As a check for robustness, for Studies 1–3 we also re-analyzed our data normalizing participants’ responses on each trial to sum to 1; all results reported here are consistent with this check.^2^ Note that these are only meaningful in asymmetric relationships, where the roles differ.

## Supplementary Material


