# Supplementary material for: Expectations of Reciprocal Generosity Are Specific to Equal Relationships
Source: Open Mind (Camb). 2026 Jun 2;10:787–807. doi: 10.1162/OPMI.a.357 (PMC13249465; doi:10.1162/OPMI.a.357)
Supplement: Supplementary file 1 [file opmi-10-787-s001.pdf]

A Supplementary Information

Contents

A Supplementary Information **1**
A.1 Deviations from preregistrations 2
A.2 Sensitivity analyses 4
A.3 Robustness checks for ceiling effects 6
A.4 Scenarios 7
A.5 Results by scenario 10
A.6 Scenario validation experiment 12
A.7 Concrete relationships in Study 3 16

A.1 Deviations from preregistrations

Overall, some experiments contained preregistered analyses grouping both higher- and lower-ranked conditions into an ‘asymmetric’ variable. We preregistered it this way to enable direct comparison with Study 1, where the conditions manipulated whether the characters were in an ‘asymmetric’ relationship or a ‘symmetric’ relationship. However, we chose not to report the comparisons with the grouped variables, for simplicity. The direct comparisons with the higher-ranked and lower-ranked conditions are themselves informative for the ‘asymmetric’ comparison because those conditions did not differ meaningfully across experiments.

Second, we also preregistered interaction contrasts (differences between expectations of precedent vs. reciprocity, between relationships). We also did not report those comparisons for simplicity, because they are also directly implied by the existence of the within-relationship contrasts that we reported.

Additionally, we computed Bayes factors (BF_{01}) for theoretically important null findings, which were not preregistered.

Finally, the robustness checks reported below were also not preregistered: the within-subject z-scored model and the cumulative link mixed model for Study 1 (Section A.3), and the analyses re-normalizing each participant’s responses on each trial to sum to 1 for Studies 1–3 (main text footnote).

The deviations for the specific experiments are reported below. The results for the updated analyses are consistent with the original ones, and the conclusions remain unchanged.

Study 1

In our preregistration, we specified that the main analyses would include only the non-control levels of each factor (excluding the ‘no relationship information’ and ‘no future interaction’ conditions). We indicated that secondary analyses would incorporate all levels. This approach was initially chosen to simplify the interpretation of interaction terms.

However, in the analyses presented in the main text, we used full models including all levels of each factor. We also employed a (non-preregistered) Type III ANOVA to test interaction effects, which we later learned was a more effective method for evaluating overall interactions and accounting for variance associated with control conditions. To test our specific hypotheses, we conducted pairwise comparisons between estimated marginal means.

The final sample was 59 participants rather than the preregistered 60. No participants met our exclusion criteria.

Study 2

The link between experimental conditions and dominance and prestige was not preregistered, nor were the analyses involving relative costs and benefits as predictors of participants’ responses.

The preregistration predicted a monotonic ordering of expected repetition across lower-, higher-, and equal-status relationships (lower $>$ higher $>$ equal). Our finding of moderate evidence for no difference between lower- and higher-ranked conditions ($BF_{01} = 11.79$) diverges from this strict ordering, although the broader pattern (hierarchical $>$ equal) is consistent with the preregistered prediction.

Study 3

The analyses considering the additional relationship features from Cheng et al. [1] were not preregistered.

Study 4

The preregistered model examining expectations for precedent versus reciprocity did not include a term for whether the first action aligned with expectations. Instead, it treated consistent and inconsistent actions together. For the final reported analyses, we added this predictor and its interaction with the initial action, enabling us to directly compare the consistent and inconsistent cases.

In addition, the specific contrast testing whether higher- or lower-status characters were more likely to repeat a generous action (replicating Study 2) was not preregistered. The robustness analyses predicting the results of Studies 2 and 3 using Study 4 data, as well as the analysis incorporating features of individual relationships, were also not preregistered.

Study 5 and 6

The preregistered model examining whether relationship predicted social interaction sequence included only trials after a successful coordination. In the reported analyses, we incorporated all available data and also added an interaction with a first successful coordination. This more complex model allowed us to capture additional variance and explore differences across both successful and unsuccessful coordination, in a single regression.

For both studies, the preregistered target sample was 160 participants. The actual samples were 156 (Study 5) and 162 (Study 6), reflecting minor variation in recruitment on Prolific. Sensitivity analyses (Section A.2) confirm that the achieved samples were well powered for the observed effect sizes.

A.2 Sensitivity analyses

Sample sizes for Studies 1–4 were determined in advance based on visual inspection of pilot data and kept consistent across studies. The same approach was used for Studies 5 and 6. Although we did not conduct formal power analyses prior to data collection, we report sensitivity analyses below to characterize the minimum effect sizes detectable with our achieved samples.

For Studies 1–3 (linear mixed models), the primary tests are within-subject pairwise contrasts (e.g., Precedent vs. Reciprocity within each relationship condition). Each participant contributes one mean for each condition (averaged across scenarios assigned to that condition), so the contrast is analogous to a paired comparison with N equal to the number of participants. Using the `pwr` package in R, we computed the minimum detectable Cohen's d at 80% power ($\alpha = 0.05$) for each study via `pwr.t.test` with `type = "paired"`.

This approach treats the contrast as a simple paired t-test, which does not account for the mixed-model structure (crossed random intercepts for participant and scenario, multiple scenarios per condition). Because the actual mixed models borrow strength across scenarios and account for scenario-level variance, the true power is likely higher than what the paired t-test implies, making these sensitivity estimates conservative. Note also that the assignment of scenarios to relationship conditions was counterbalanced across participants, so the Precedent vs. Reciprocity contrast is fully within-scenario (each participant rates both for every scenario they see), whereas contrasts across relationship conditions (e.g., Higher vs. Lower) involve different scenarios in each condition. The sensitivity estimates apply most directly to the within-scenario contrasts.

Table S1: Minimum detectable effect sizes at 80% power.

Study	N	Min. detectable d	Approx. min. OR
1	59	0.37	–
2	59	0.37	–
3	57	0.38	–
4	113	0.27	1.62
5	153	0.23	1.51
6	158	0.22	1.50

For Studies 1–3, the minimum detectable effect sizes fall between the conventional thresholds for small ($d = 0.20$) and medium ($d = 0.50$) effects. The observed effect sizes for the primary contrasts in asymmetric relationships are substantially larger than these thresholds (e.g., $d = 1.30$ for the Precedent vs. Reciprocity contrast in asymmetric relationships in Study 1), indicating that the studies were well powered for these effects. However, the preference for reciprocity over precedent in equal relationships was smaller ($d = -0.19$ in Study 1, 0.36 in Study 2, 0.18 in Study 3) and falls near or below the detectable threshold. The key finding of this paper is the strong shift toward precedent in hierarchical relationships, for which the studies were well powered.

For Studies 4–6 (logistic mixed models), precise sensitivity estimates would require simulation-based power analysis that accounts for the binary outcome, crossed random effects, and condition-specific base rates. As a simple approximation, we treat each participant's proportion of precedent-following choices per condition as a continuous outcome and apply the same paired-comparison framework. We convert the resulting d to an approximate odds ratio using $OR \approx \exp(d \cdot \pi/\sqrt{3})$ [2]. This approximation does not account for the mixed-model structure or the discrete nature of individual binary trials, but provides a rough sense of the minimum detectable effect size. The observed primary effects are larger in magnitude than these approximate minimum detectable effects (minimum detectable $|\log \mathrm{OR}| \approx 0.41$–$0.48$; e.g., for the

reciprocity effect in Study 5, $\mathrm{OR} = 0.42$, so $|\log \mathrm{OR}| = 0.87$). The Bayes factors reported in the main text additionally address the interpretability of the null findings in these studies.

A.3 Robustness checks for ceiling effects

In Study 1, the precedent rating in asymmetric relationships (M = 5.25 on a 7-point scale) raised concerns about potential ceiling effects. We addressed this with two robustness checks: a within-subject z-scored model and a cumulative link mixed model (ordinal regression). We also note that ceiling effects, if present, would work against our primary finding: compression at the high end of the scale would attenuate the Precedent vs. Reciprocity difference in asymmetric relationships, meaning the true effect is likely larger than what we observe.

Z-scored model

We z-scored each participant's Likert ratings within subject (subtracting each participant's mean and dividing by their standard deviation across all trials). This removes between-subject differences in scale usage and reduces the influence of ceiling or floor effects on the group-level contrasts.

We refit the primary linear mixed-effects model using the z-scored ratings as the dependent variable. The pattern of results was unchanged. The relationship × next-interaction interaction remained significant ($F(4,3155) = 68.95$, $p < .001$). In asymmetric relationships, participants expected precedent over reciprocity (ΔM = 1.00 [0.89, 1.11], $t(3095) = 17.74$, $p < 0.001$). In symmetric relationships, participants expected reciprocity over precedent (ΔM = −0.18 [−0.29, −0.07], $t(3095) = -3.12$, $p = 0.002$). With no relationship information, neither was preferred (ΔM = 0.07 [−0.04, 0.18], $t(3095) = 1.28$, $p = 0.200$).

Ordinal model

As a further check, we fit a cumulative link mixed model [CLMM; 3] that treats the Likert ratings as ordinal rather than continuous, making no assumptions about normality or equal spacing of scale points. The model included the same fixed effects (next interaction × relationship) and random intercepts (participant and scenario) as the primary model. All four interaction terms were significant ($z = 4.38$, $p < 0.001$; $z = -3.66$, $p < 0.001$; $z = 10.23$, $p < 0.001$; $z = -5.75$, $p < 0.001$), confirming that the relationship × next-interaction pattern does not depend on the assumption of a continuous, normally distributed outcome.

These results indicate that the primary findings are not driven by ceiling effects or distributional assumptions.

A.4 Scenarios

Label	Scenario
architects	Consider Sonia and Liana, who work as architects at the same company. They work on the same team, where they work together on their designs. Sonia meticulously double-checks finished designs before they send them out.
babysitting	Consider Ryan and John, who both have two children under 10 years old. This week, Ryan drops their children off John's house, where John volunteers to watch Ryan's children so Ryan can have a date night.
coffee	Consider Andrew and Bob, who work in the same field. They meet for coffee. Bob buys and brings coffee to the meeting for both of them.
concerts	Consider Kevin and Allen. They go to a concert together, and Kevin pays for the tickets.
conversation	Consider Alice and Angela, who work at the same company. They have a one-hour meeting. Alice spends most of the one-hour meeting talking, while Angela spends most of the one-hour meeting listening and asking questions.
family meals	Consider Sue and Martha, who are part of the same family. The whole family gets together for a religious holiday, and gathers at Sue's house. Sue cooks all the food, serves it, and then cleans up after dinner. Martha is very appreciative, and gives Sue a big hug and a warm thank you.
feedback	Consider Taylor and Samantha, who are both fiction writers living in the same city. They frequently attend the same group writing workshops. Samantha shares work in progress, and Taylor gives her feedback on it.
fundraising	Consider Caroline and Bella, who both frequently run for charity. They run a race together and have to raise money for their charity to enter the race. Caroline takes the lead on the advertising and fundraising, contacting and following up with her friends to ask them if they would like to donate to their charity.
gifts	Consider two families, the Lee family and the Min family. Each member of the Lee family gives the Min family a precious and expensive gift.
groceries	Consider Dylan and Caleb, who live on the 5th floor of a walkup in Manhattan, with a steep narrow staircase. Each of them comes home from the grocery store with many heavy bags. Caleb helps carry Dylan's groceries up the stairs.
group project	Consider Maxwell and Ralph, two people at the same company who are assigned to work together on the same project. They have a meeting where Maxwell contributes most of the ideas, delegates tasks to Ralph, and decides what to do next.
housemates	Consider Sarah and Anne, who both live in the same old building. Sarah notices something damaged or missing or needed for the house and writes it on a list. Anne completes all of the tasks on the list.
meeting location	Consider Ethan and Benjamin, who work at the same Boston-area nonprofit. Ethan walks to Benjamin's office, where they have a meeting.
meeting prep	Consider Chloe and Victoria, who work at the same news station. They have a meeting where Chloe prepares the agenda, and books the meeting room.
police	Consider Justin and Jarred, who are police officers on the same beat. Justin goes to the office at 6am to do the day's paperwork, so that Jarred can arrive later and go straight to their shared patrol car.
restaurant	Consider Avery and Grace, who want to go out to eat. There are two restaurants near their work: a Chinese restaurant, and an Indian restaurant. Avery prefers the Chinese restaurant, while Grace prefers the Indian restaurant. They go to the Chinese restaurant.
scheduling	Consider Matt and William, who want to have a joint meal at an Italian restaurant near their workplace. Matt prefers lunches, because Matt likes to have a free schedule after work. William prefers dinners because William prefers not to break up the work day by traveling somewhere after lunch. They get dinner on Wednesday at 7pm.
weekend	Consider Abby and Evelyn, who want to spend time together during weekends. Abby prefers waking up early to do intense outdoor activities, while Evelyn prefers sleeping in, getting brunch, and walking around the city. This weekend, Abby and Evelyn wake up early both days to do intense outdoor activities.

Table S2: Scenarios for Study 1-4.

**Label**	**Equal**	**Hierarchical**
architects	Co-lead architects	Lead architect and junior designer
babysitting	Religious group co-members	Religious group leader and follower
coffee	Peers with the same roles	Advisor/mentor and advisee/mentee
concerts	Friends	Older brother by ten years who helps younger brother with many tasks
conversation	Coworkers who started around the same time and have the same roles	Boss and employee
family meals	Cousins	Aunt and niece
feedback	Equally experienced writers	Established writer and graduate student who hasn't yet published her work
fundraising	Training partners	High-school runner and coach
gifts	Next-door neighbors whose children are the same ages	A pair of established professionals, and two graduate-student parents and their toddler
groceries	Freshman roommates	Residential advisor and student
group project	Co-interns on the same project	Senior developer and intern
housemates	Roommates who split rent and bills equally	Owner and renter
meeting location	Mid-level employees	Manager and employee
meeting prep	Co-writers on the same show	News anchor and assistant
police	Officers of the same rank who are partners on the same shift	Sergeant and regular officer
restaurant	Vice presidents at the same company	Vice president of operations and CEO
scheduling	Coworkers	Established in field with expertise and connections, and advice-seeker
weekend	Cousins who are about the same age	Older cousin who is also legal guardian

Table S3: Concrete relationships used in Study 3.

Label	Scenario	Choices
gift	After participating in an important communal event, it is customary for one person to offer a meaningful token to the other. The other person receives the token as a sign of shared experience and mutual respect.	Give a token / Accept a token
meal	When two people share a ceremonial meal, one person must serve the food while the other receives it first.	Serve the food first / Wait to be served
meeting place	Some days, a pair of people is assigned to check on the fields furthest from the village, which means leaving for a long walk early in the morning. You know that there is a meeting place closer to your house, and a meeting place closer to your partner's house.	Go to the place closer to your partner's house / Go to the place closer to your own house
object carrying	At the end of a ritual, the sacred objects must be returned to their keeper. Someone must carry the heavier object and someone must carry the lighter object.	Carry the heavier object / Carry the lighter object
song preference	Each week ends with a village event where pairs of people perform in front of the community. One song you know has a great solo part for your instrument, while the other song highlights your partner's instrument.	Choose the song that's better for your partner's instrument / Choose the song that's better for your own instrument
task checking	The people in the village are paired up, to work together on a series of tasks for the village. It is important that each task is done correctly and that no mistakes are made, so one person needs to do a detailed check.	Perform the detailed check / Wait for your partner to do a detailed check

Table S4: Scenarios for Study 5-6.

A.5 Results by scenario

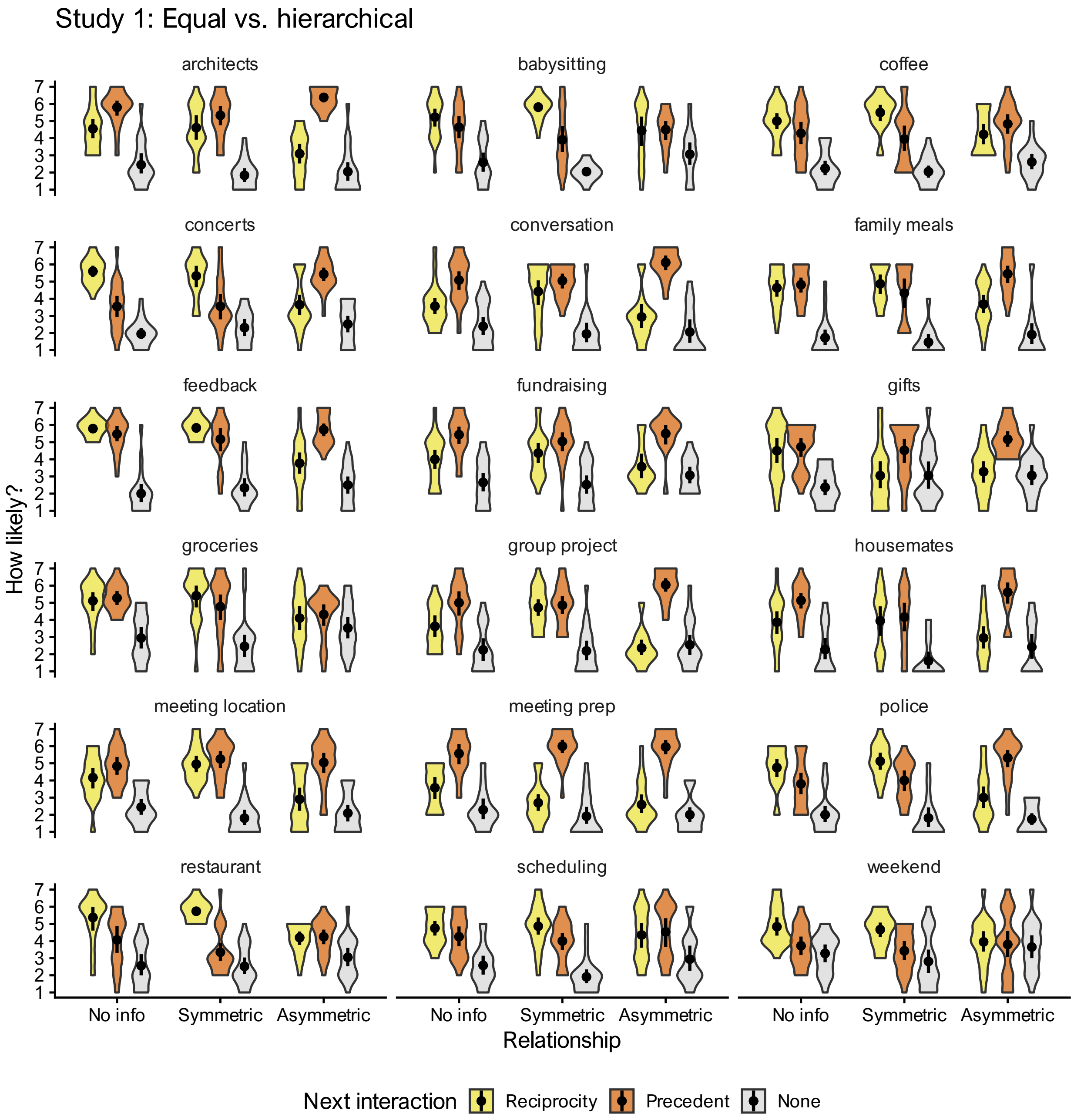

Figure S1: Study 1 results by scenario. Error bars are bootstrapped 95% confidence intervals.

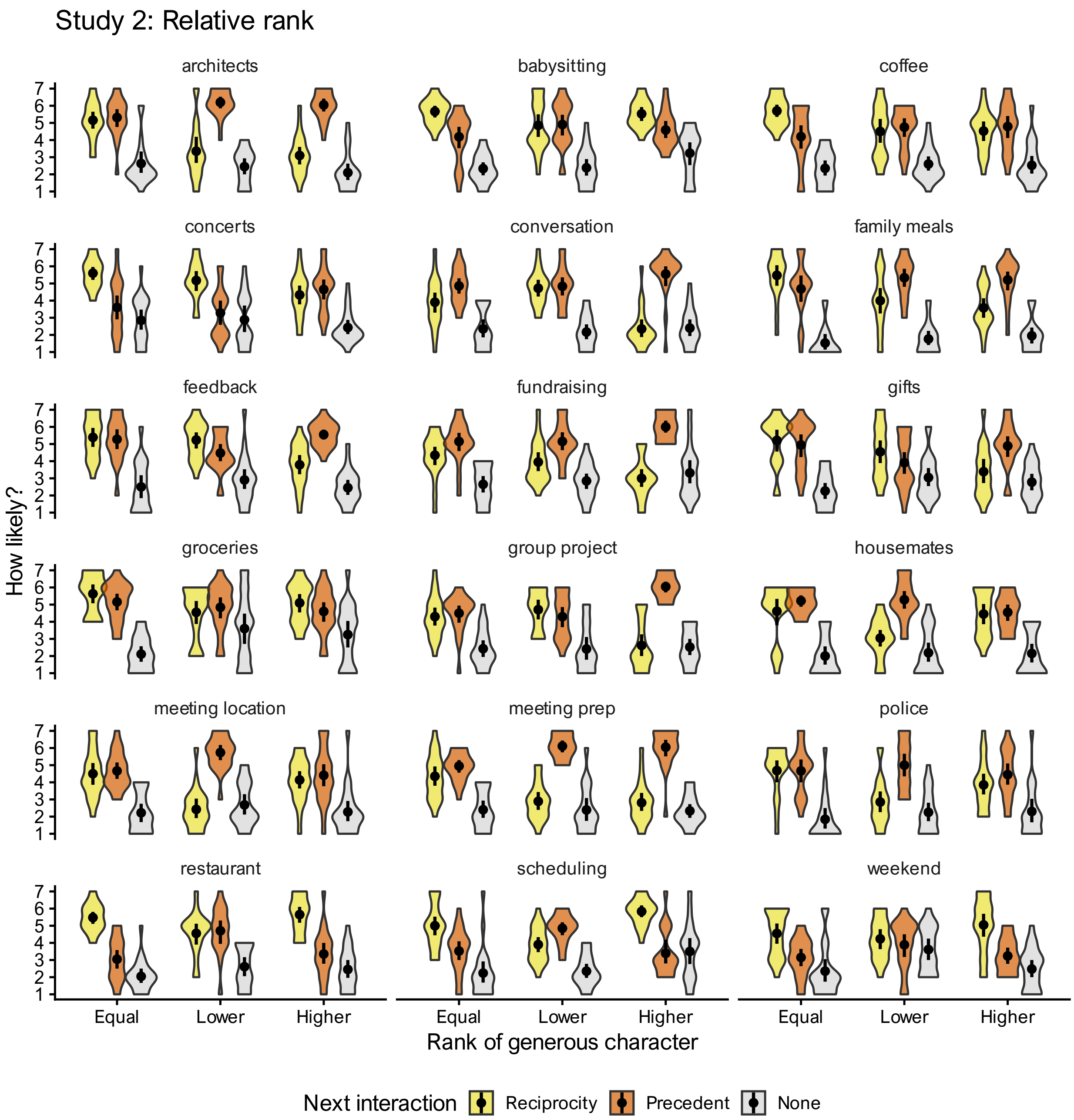

Figure S2: Study 2 results by scenario. Error bars are bootstrapped 95% confidence intervals.

A.6 Scenario validation experiment

Generous behavior has been defined as an act where one person incurs a cost, to benefit another [4], and these measures have been shown to affect adult and children's evaluations of generosity [5, 6]. Thus, to test whether there is separable variance in the scenarios that could explain variance in the actual experiments, we ran two experiments measuring the relative cost and benefit ($N = 58$ for benefit, $N = 59$ for effort, Supplementary Figure S3) between the two people interacting in each scenario.

In each experiment, we showed participants all 18 scenarios, each scenario displaying one generous action that one character performs for the other. In the 'benefit' experiment, we asked participants how much each of the two characters in the scenario benefits from the interaction, compared to if they don't interact. In the 'effort' experiment, we asked participants how much effort each character puts into the interaction, compared to if they don't interact. Thus, participants answered two questions on each trial, corresponding to the two characters in the scenario, each using a 7 point Likert scale ('not at all' (1) - 'extremely' (7)).

The scenarios in Supplementary Figure S3 are ordered based on the differential benefit between the two people interacting. In general, people expect the recipient of the generosity to benefit more than the generous actor, and that the generous actor puts in more effort than the recipient (Supplementary Figure S3a). Additionally, relative benefit generally corresponds to relative effort (scenarios where the generous actor puts in relatively more effort also correspond to the scenarios where they benefit less) (Supplementary Figure S3a). Some scenarios are more uneven in either benefit or effort: For example, in the 'restaurant' scenario, participants expect that the generous actor (who offers to go to the recipient's preferred restaurant) puts in comparatively less effort, compared to how much the recipient benefits from the interaction.

Overall, the results of these experiments suggest that, in general, our scenarios display uneven benefit or uneven effort (one character puts in more effort, or benefits more, than the other), and that they cover a wide range of benefit and effort.

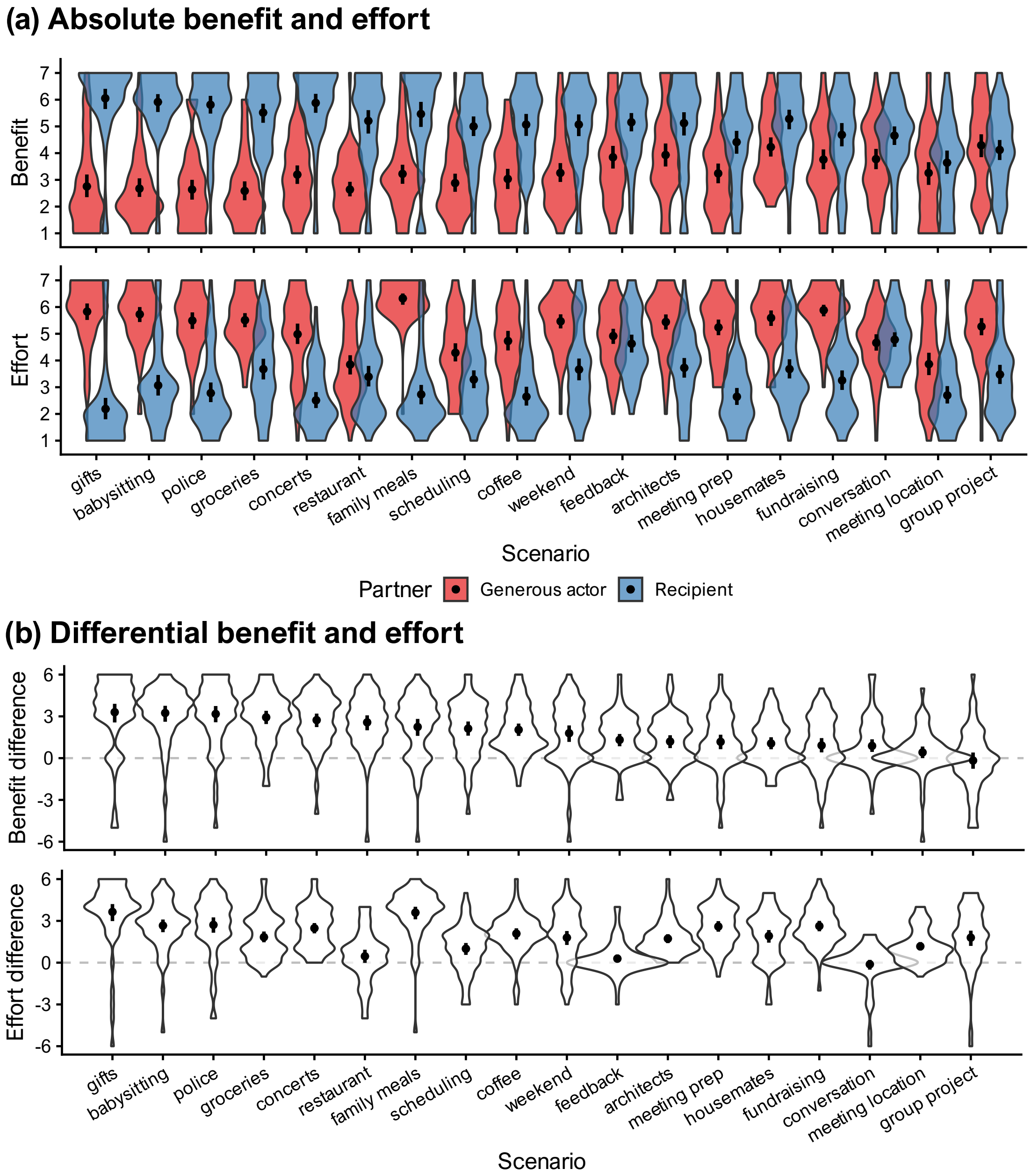

Figure S3: Results from experiments measuring differential benefit and effort. Error bars are bootstrapped 95% confidence intervals.

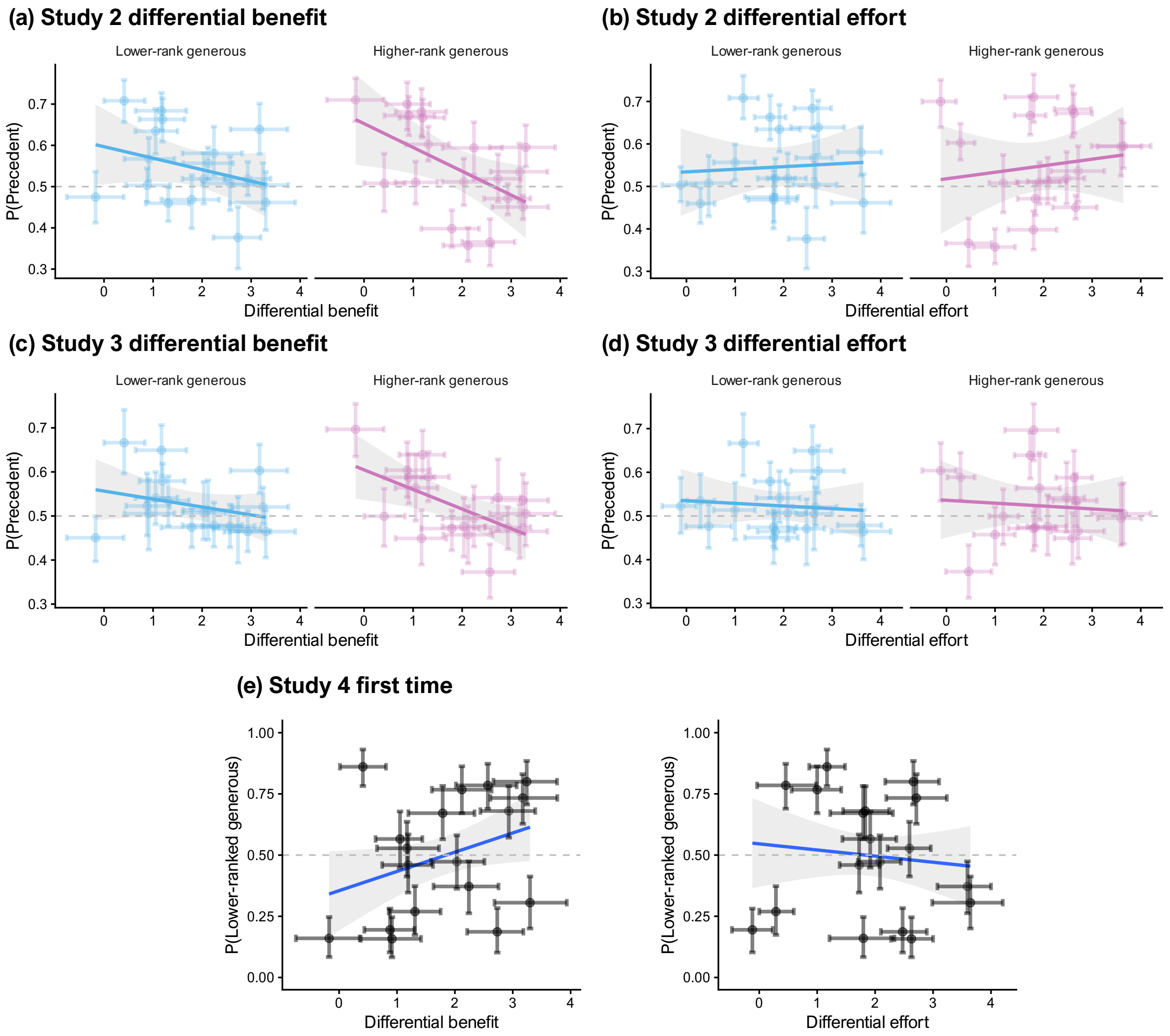

Figure S4: Predicting results in Study 2, 3, and 4 using measured differential benefit and effort. Error bars are bootstrapped 95% confidence intervals.

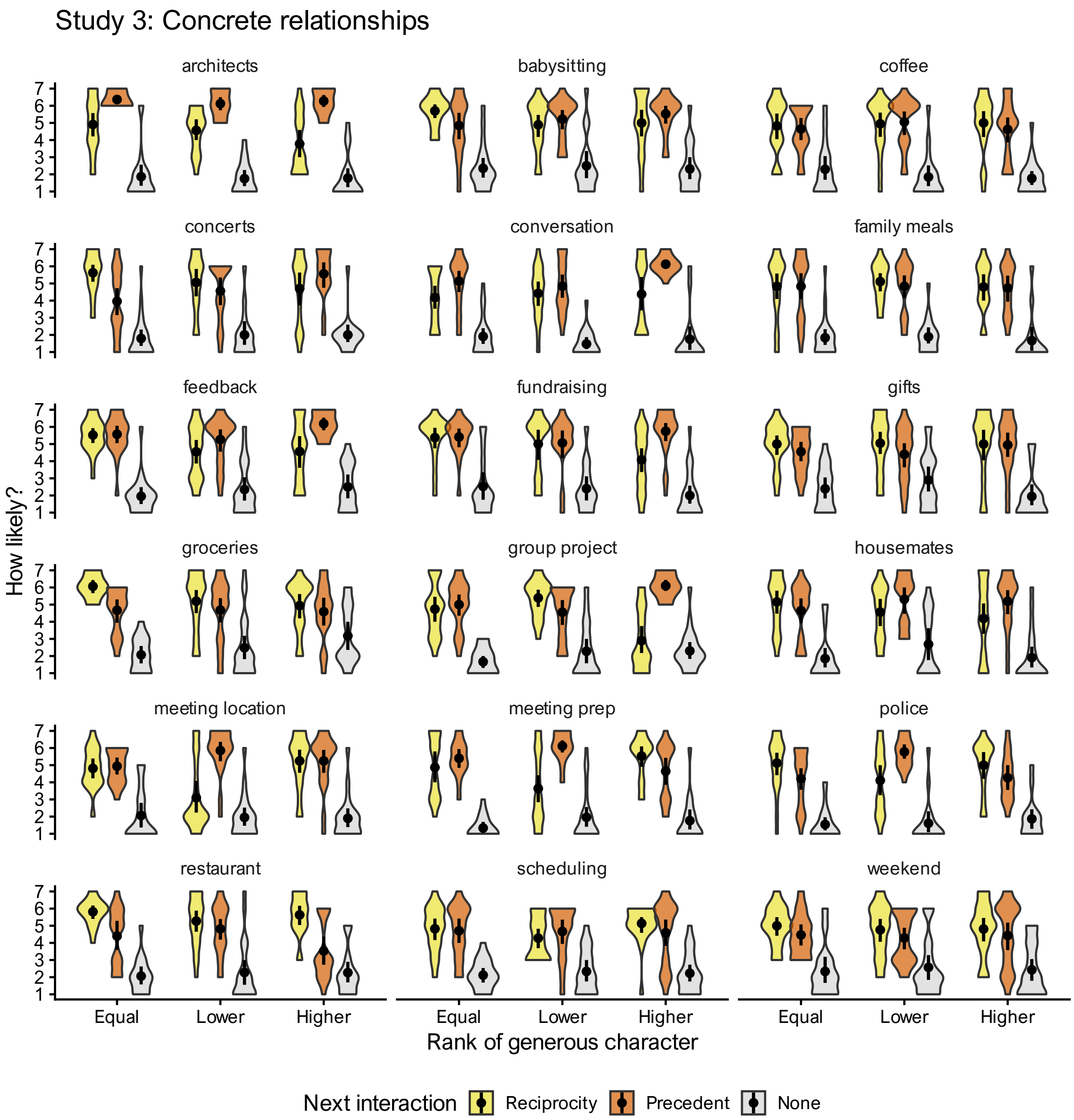

Figure S5: Study 3 results by scenario. Error bars are bootstrapped 95% confidence intervals.

A.7 Concrete relationships in Study 3

Since Study 3 involved concrete relationship labels (e.g., boss-employee), we explored whether additional variance could be explained by including structural features of these relationships. We incorporated continuous dominance and prestige scores derived from a large-scale study [1]. In the study, participants judged different kinds of concrete relationships on 30 different features, summarized from 15 different theories in the prior literature; the scores were continuous values between 0 and 100. If the exact relationship was not in their data, we picked the best intuitive match based on the features of the relationship in the scenario. We computed dominance and prestige scores by aggregating the features based on the definitions of dominance and prestige as described in Henrich and Gil-White [7]: The dominance score was obtained by averaging the scores for "Coercion" and "Conflict," and the prestige score was obtained by averaging the scores of "Importance for society," "Importance for individuals," and "Information transmission." Dominance and prestige were not significantly correlated with each other (Pearson's $r = 0.23$, $t(34) = 1.38$, $p = 0.176$) (Supplementary Figure S6c), nor with the initial expectations for who should be generous (dominance: Pearson's $r = 0.18$, $t(16) = 0.72$, $p = 0.484$; prestige: Pearson's $r = -0.13$, $t(16) = -0.54$, $p = 0.599$) (Supplementary Figure S6a), suggesting that they captured distinct dimensions. Indeed, adding these dominance and prestige scores[1] improved model fit (likelihood-ratio test $\chi^2(4) = 17.43$, $p = 0.002$). However, there were no significant main effects of dominance nor prestige (dominance: $b = 5.09 \times 10^{-4}$, $t(14.0) = 0.69$, $p = 0.499$; prestige: $b = 1.73 \times 10^{-3}$, $t(14.0) = 1.72$, $p = 0.107$) (Supplementary Figure S6b). We did find a significant interaction involving dominance: in relationships with higher dominance scores, observing a lower-ranked individual acting generously (i.e. presumably confirming one's beliefs about a dominant relationship) increased the likelihood that participants expected the same individual to act generously again (dominance $\times$ observed first action interaction: $b = 1.39 \times 10^{-3}$, $t(658.6) = 3.11$, $p = 0.002$).

[1]Formula: `expected_next_3 ~ (dominance_score + prestige_score + expected_first_4) * observed_first + (1 | participant) + (1 | scenario)`

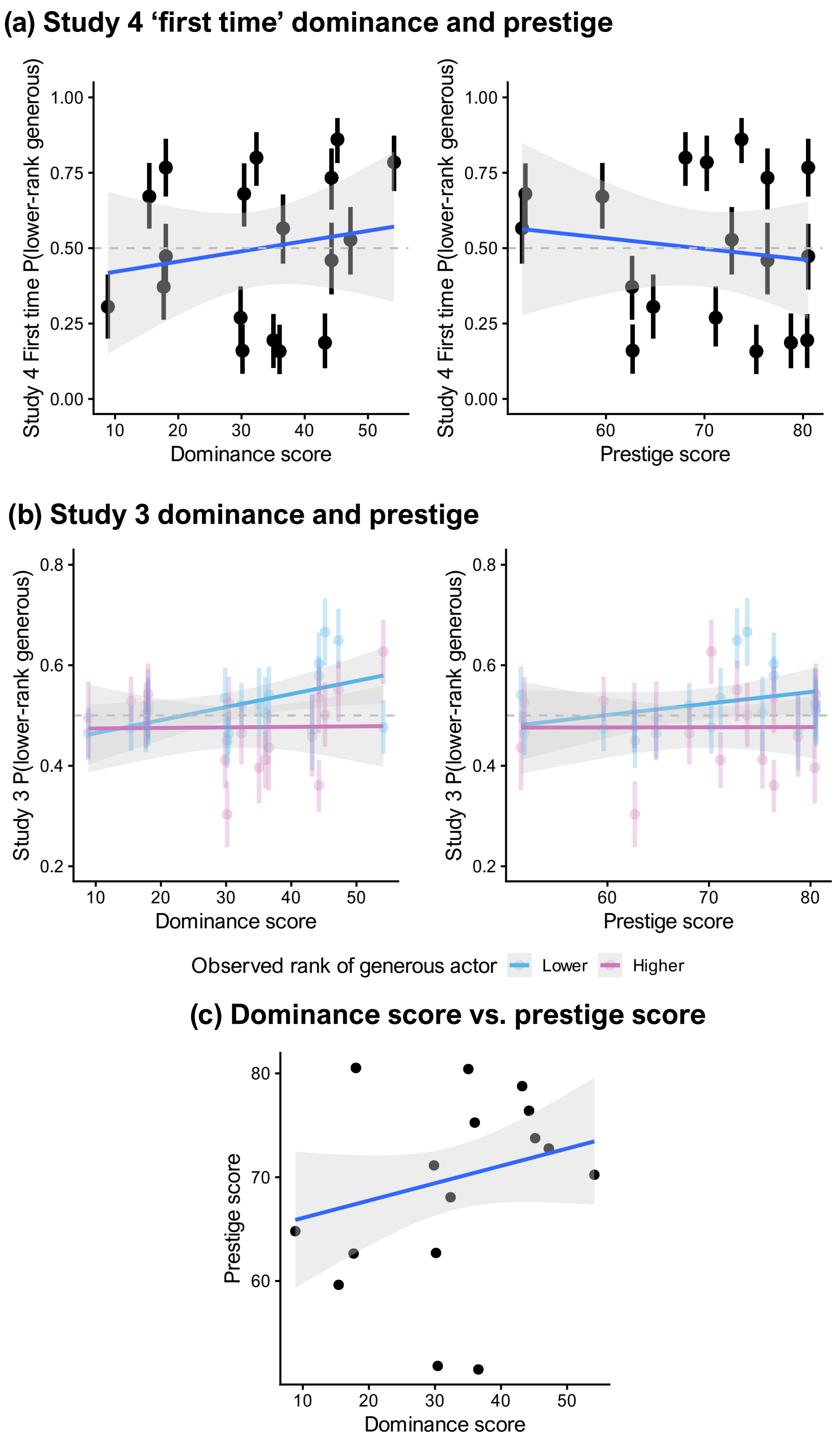

Figure S6: Predicting the results of Study 3 and 4 using dominance and prestige scores derived from a large-scale study. Error bars are bootstrapped 95% confidence intervals.

References

[1] Xi Cheng, Haroon Popal, Huanqing Wang, Renfen Hu, Yinyin Zang, Mingzhe Zhang, Mark A Thornton, Yina Ma, Huajian Cai, Yanchao Bi, et al. The conceptual structure of human relationships across modern and historical cultures. *Nature Human Behaviour*, pages 1–14, 2025.

[2] Susan Chinn. A simple method for converting an odds ratio to effect size for use in meta-analysis. *Statistics in Medicine*, 19(22):3127–3131, 2000.

[3] Rune Haubo Bojesen Christensen. *ordinal – Regression Models for Ordinal Data*, 2019.

[4] Ernst Fehr and Urs Fischbacher. The nature of human altruism. *Nature*, 425(6960):785–791, 2003.

[5] Jessica A Sommerville, Elizabeth A Enright, Rachel O Horton, Kelsey Lucca, Miranda J Sitch, and Susanne Kirchner-Adelhart. Infants' prosocial behavior is governed by cost-benefit analyses. *Cognition*, 177:12–20, 2018.

[6] Mia Radovanovic, Tim Wei-Ting Chao, Emily Onyshko, Quang David Tri Huynh, Yang Leona Liu, and Jessica A Sommerville. Not just if, but how much: Children and adults use cost and need to make evaluations about generosity across contexts. *Cognition*, 238:105533, 2023.

[7] Joseph Henrich and Francisco J Gil-White. The evolution of prestige: Freely conferred deference as a mechanism for enhancing the benefits of cultural transmission. *Evolution and human behavior*, 22(3): 165–196, 2001.
